# A case report of traumatic ulcerative granuloma with stromal eosinophilia (TUGSE) in a 21‐year‐old

**DOI:** 10.1002/ccr3.3188

**Published:** 2020-09-09

**Authors:** Tufayl Ahmed Hannan, Muhammed Umer, Labib Syed, Mohamed Areeb Anis‐Alavi

**Affiliations:** ^1^ Barts and The London School of Medicine and Dentistry London UK

**Keywords:** carcinoma, dentistry, geriatrics, maxillofacial, TUGSE, Ulceration

## Abstract

We present an unusual case of a persistent solitary left palatoglossal ulcer with no history of trauma or associated risk factors. A TUGSE lesion, which mimics that of malignancy, must always be noted as a differential even in risk factor absence.

## Graphical abstract

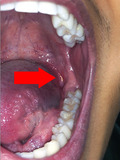

## 1 BACKGROUND

Traumatic ulcerative granuloma with stromal eosinophilia (TUGSE) is a rare, benign condition affecting the oral cavity primarily the anteroventral and dorsal surface of the tongue.^1^

It is a self‐limiting and self‐healing condition; however, the rapid onset, slow healing, and rolled borders mean it is often mistaken for a sinister pathology such as an oral squamous cell carcinoma.^2^ A lack of production of transforming growth factors by eosinophils in chronic oral ulcers has been observed to decrease the rate of healing and thus may explain the delayed healing in TUGSE.^3^

It often presents as a solitary asymptomatic or painful ulcer and may last from weeks to months.^4^ It is most commonly diagnosed in the 5th decade of life, but can present at any age and is referred to as Riga‐Fede disease if diagnosed in neonates or infants.^5^, ^6^ The major etiology for TUGSE is trauma; however, some of the other possible causes include sharp tooth margins, ill‐fitted dentures, and incisional biopsies.^3^

## 2 CASE REPORT

A 21‐year‐old male patient presented to the oral maxillofacial clinic after urgent referral by his general practitioner on the 2‐week wait pathway. He presented with a 4‐week history of sore throat and mild odynophagia radiating to the left submandibular region. The patient further mentioned the presence of a solitary white plaque on the lower left palatoglossal region implying possible leucoplakia. There was no history of local trauma to the affected area. The patient had a previous root canal with a composite semi‐crown on the upper right central incisor 4 years ago. Otherwise, his past medical, drug, and social history was unremarkable.

**FIGURE 1:**
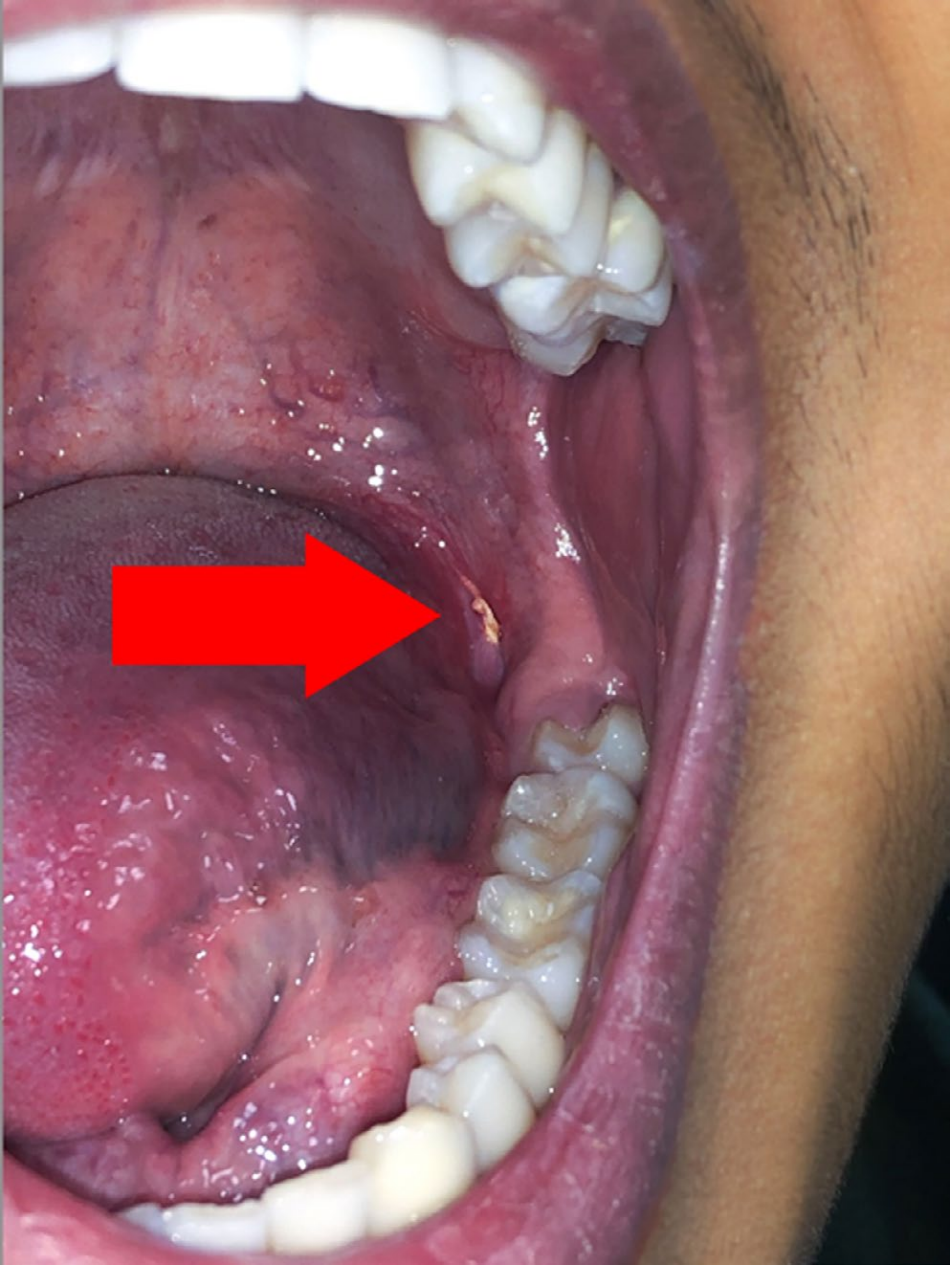
Presentation of the lesion postexcision and biopsy. Red arrow indicating the stitches can be seen on the left palatoglossal region

On clinical examination, there was a flat, 2 mm poorly healing solitary lesion with an indurated base located in the left palatoglossal fold region posterior to tooth 38, appearing clinically like a TUGSE. Provisional diagnosis of a TUGSE was made with consideration of a differential of squamous cell carcinoma, aphthous stomatitis, and necrotizing sialometaplasia; thus, a subsequent excisional biopsy under local anesthetic for histopathological examination was arranged, and the outcome of the procedure is depicted in Figure 1.

Two months after diagnosis, there was no recurrence of ulceration, pain, or discomfort. However, the mucosa appeared to show signs of microtrauma but was healing well.

## 3 DISCUSSION

Traumatic ulcerative granuloma with stromal eosinophilia is a rare, benign condition with an unclear nature, cause, and physiological mechanism. The pathogenesis of TUGSE is currently unknown. However, one of the theories postulates that trauma causes ulceration which allows influx of microorganisms, toxins, and foreign debris into the surrounding tissue. A severe inflammatory response is triggered secondary to mast cell‐eosinophil reaction which recruit eosinophils and cause further damage by exacerbating inflammation locally.^1^

Traumatic ulcerative granuloma with stromal eosinophilia is difficult to diagnose clinically; therefore, diagnosis must be based on histological and clinical reports. Histologically, a deep ulceration is observed, and overlying epithelium is covered with fibrinopurulent membrane. The ulcerated area of the lesion which can penetrate the muscle fibers contains eosinophils, neutrophils, and fibroblasts.^7^ Some lesions are infiltrated with polymorphic lymphocytes, and immunohistochemistry analysis of these lesions shows presence of lymphocytes with the following: CD3+, CD2+, CD4+, CD8‐, CD5+, CD7‐.^8^

Traumatic ulcerative granuloma with stromal eosinophilia commonly occurs in elderly patients with a history of trauma and variations in oral anatomy. However, this case presented with none of these factors aside from the expected presentation of a solitary ulcer. Given that TUGSE presents very similar to oral cancer, it is vital as diagnosticians we include this as an important differential when patients present with signs and symptoms mimicking oral cancer.

Several treatment options are available for TUGSE which include conservative management, corticosteroids, antibiotics, and cryotherapy but the recommended mainstay treatment is surgical excision, and patients recover well and rarely present with lesion recurrence.^9^

## 4 CONCLUSION

In summary, diagnosis of TUGSE is achieved through clinical and histopathological findings. The concrete pathogenesis for this remains unclear particularly if the patient presents in the absence of risk factors or a history of trauma as in this unusual case in a young patient. However, excision and reassurance was effectively delivered to this patient and the requirement for radical surgery was not necessary.

## Conflict of Interest

We have no conflicts of interest to disclose.

## Author Contributions

TAH: Reviewed previously written case reports in the Clinical Case Reports Journal to gain an idea of the optimal layout and structure for the manuscript, obtained and analyzed patient data alongside Umer, with relevance to the diagnosis of TUGSE, received explanation of TUGSE pathophysiology and mechanisms to draft a relevant case report for Umer to produce formally, contributed to the drafting of the manuscript with particular attention to the case report and conclusion of the results, compiled the final revised version using the feedback provided by the co‐authors, and finalized and updated the revision using the editorial comments alongside Syed. LS: Alongside Hannan reviewed previously written reports to gain an insight into the requirements by the Clinical Case Reports Journal on the internal contents of the manuscript, reflected on the sections produced by the co‐authors to draft the discussion of the paper on the importance of the case and TUGSE, conducted revision of the manuscript applying comments for grammatical and scientific improvements, and finalized and updated the revision using the editorial comments alongside Hannan. MU: Formally drafted the patient case report using Hannan's notes enabling the partner authors to utilize the patient details, learned and explained the pathophysiological mechanisms of TUGSE alongside Anis‐Alavi which aided case report drafting, contributed to the drafting of the manuscript with particular attention to the case report, and conducted revision of the manuscript applying comments for grammatical and scientific improvements. MAA‐A: Learned and explained the pathophysiological mechanisms of TUGSE with Umer to Hannan who collected patient details, composed the background section of the case report due to having researched into the condition, and conducted revision of the manuscript applying comments for grammatical and scientific improvements.
